# An eHealth Program for Patients Undergoing a Total Hip Arthroplasty: Protocol for a Randomized Controlled Trial

**DOI:** 10.2196/resprot.9654

**Published:** 2018-06-12

**Authors:** Rosemary Saunders, Karla Seaman, Catherine Ashford, Trudy Sullivan, Judith McDowall, Lisa Whitehead, Beverley Ewens, Kathryn Pedler, Karen Gullick

**Affiliations:** ^1^ School of Nursing and Midwifery Edith Cowan University Joondalup Australia; ^2^ Hollywood Private Hospital Nedlands Australia; ^3^ Department of Preventive and Social Medicine University of Otago Dunedin New Zealand

**Keywords:** hip replacement, education, Web-based platform, eHealth program, rehabilitation, economic evaluation

## Abstract

**Background:**

Total hip arthroplasty is an effective surgical procedure commonly used worldwide for patients suffering the disabling effects of osteoarthritis when medical therapy is unsuccessful. Traditionally pre- and postoperative information for patients undergoing a hip arthroplasty has been provided by paper-based methods. Electronic health (eHealth) programs to support individualized patient education on preoperative preparation, in-patient care, and home rehabilitation have the potential to increase patient engagement, enhance patient recovery, and reduce potential postoperative complications.

**Objective:**

The aim of this study is to compare the addition of an eHealth program versus standard care for pre- and postoperative education on patient outcomes for primary total hip arthroplasty.

**Methods:**

One hundred patients undergoing a primary elective total hip arthroplasty will be recruited from a metropolitan hospital in Western Australia to participate in a 6-month parallel randomized control trial. Participants will be randomized to either the standard care group (n=50) and will be given the education booklet and enrolled to attend a 1-hour education session, or the intervention group (n=50), and will receive the same as the standard care plus access to an eHealth program titled “My Hip Journey.” The eHealth program encourages the patient to log in daily, from 2 weeks prior to surgery to 30 days postsurgery. The information on the platform will be aligned with the patient's individual surgical journey and will include exercises to be completed each day for the duration of the program. The primary outcome measure is the Hip Dysfunction and Osteoarthritis Outcome Score, version LK 2.0. Secondary outcome measures include the EuroQoL EQ-5D-5L, a 5-level 5-dimension quality of life measure, and the Self-Efficacy for Managing Chronic Disease Scale. Data will be collected at pre-admission (presurgery) and at 6 weeks, 3 months, and 6 months postsurgery. A patient satisfaction survey will be completed 6 weeks postsurgery and Web-based analytics will be collected 6 months postsurgery. A cost-effectiveness analysis, using the intention-to-treat principle, will be conducted from the hospital’s perspective.

**Results:**

Enrollment in the study commenced in January 2018 with recruitment due for completion towards the end of the year. The first results are expected to be submitted for publication in 2019.

**Conclusions:**

The outcomes and cost of using an eHealth program to support a patient’s recovery from a hip arthroplasty will be compared with standard care in this study. If the eHealth program is found to be effective, further implementation across clinical practice could lead to improvement in patient outcomes and other surgical areas could be incorporated.

**Trial Registration:**

Australian New Zealand Clinical Trials Registry (ANZCTR) ACTRN12617001433392; https://www.anzctr.org.au/Trial/Registration/TrialReview.aspx?id=373657&isReview=true (Archived by WebCite at http://www.webcitation.org/6yzoTuggx).

**Registered Report Identifier:**

RR1-10.2196/9654

## Introduction

Osteoarthritis is a major disabling joint disorder worldwide, with the hip being the joint that is second most affected therefore resulting in pain, decreased function, and reduced quality of life [1]. Total hip arthroplasty (THA) is an effective surgical procedure that improves both joint function and quality of life for patients with hip osteoarthritis [2]. Internationally, there has been a significant increase in the number of THA procedures in the past ten years, and similarly each year the number of people in Australia undergoing hip arthroplasty has increased [3-4]. In Australia, in 2015, there were 44,710 hip arthroplasties (77.2% primary THA, 13.3% primary partial hip arthroplasty, and 9.6% for revision of hip arthroplasty). This represents a 2.6% increase in the number of hip arthroplasty procedures compared with 2014. Over half (59.7%) of all hip arthroplasty procedures in 2015 were undertaken in private hospitals [4].

For patients undergoing a total joint replacement, pre-admission, pre-operative, and postoperative patient education is essential. The mode of education delivery currently includes one-to-one verbal conversations, patient group sessions, educational booklets, and educational videos [5]. Many studies have explored the effects of these education programs on patient outcomes, including length of hospital stay, pain, functional abilities, knowledge, anxiety, and quality of life. A Cochrane review on pre-operative education for hip and knee replacements reported unequivocally that pre-operative education offers benefits over usual care in relation to or surgical outcomes, such as pain, function, and adverse events [6]. The review noted that pre-operative education may represent a useful adjunct with a low risk of undesirable effects. A more recent review linked pre-operative education to improved patient outcomes including lower length hospital of stay, higher rate of home discharge, lower readmission, and improved cost-effectiveness [6]. Additionally, other recent evidence not included in the Cochrane review demonstrated that the provision of pre-operative education is linked to a reduction in hospital length of stay [7-10].

An alternative forum for delivering patient education is through electronic health (eHealth). The World Health Organization defines eHealth as “the use of information and communication technologies for health” [11]. The introduction of eHealth programs to support individualized patient care at the pre-operative, peri-operative, and postoperative stages has the potential to improve patient engagement, self-care, and outcomes across the surgical pathway [12]. The implementation of eHealth programs enables a single source of credible information, which can be constantly updated as new information arises, and is accessible to all patients irrespective of geographical location. Programs can be individually tailored to the patient, provide a platform for communication with health care professionals, provide electronic reminders to prompt patients, and can be used by other health professionals and carers to provide an enhanced continuity of care [12].

The aim of this study is to compare the addition of an eHealth program versus standard care for pre- and postoperative education on patient outcomes for primary THA. The hypothesis is patients who have access to the eHealth program will have better physical functioning post-THA compared to patients receiving standard care.

## Methods

A prospective randomized controlled trial (RCT) will be conducted comparing an eHealth program to standard care for THA. Ethics approval was obtained from the participating study site (HPH505) and the University where the researchers are employed (19065).

### Study Duration

Recruitment of the trial began in January 2018 and data collection is due to be completed within 12 months.

### Setting

The setting of this study is a private metropolitan hospital in Western Australia with a focus on orthopedic surgeries. In 2017, there were a total of 848 hip surgeries performed in the hospital by 24 orthopedic surgeons. As the hospital is a private hospital, the surgeries are mostly funded through patients’ private insurance.

### Patients

Patients undergoing a primary elective THA will be invited to take part in the RCT. Patients will be screened and recruited during the usual pre-admission phone call conducted by the pre-admission nurse. Inclusion criteria are as follows: (1) the patient is undergoing a primary elective THA, (2) the patient is able to provide informed consent, (3) the patient is aged 18 years or over, and (4) there is a minimum lead-up time of three weeks prior to surgery. Exclusion criteria include the following: (1) the patient is undergoing a THA revision, (2) a bilateral THA, or (3) THA following a fractured neck of femur, (4) had a previous THA, (5) unable to converse in written or spoken English, (6) has no access to a Web-based device, and (7) has a risk assessment and prediction tool (RAPT) score less than six. The RAPT uses pre-operative patient factors of age, gender, pre-operative ambulatory distance, use of gait aid, community support, and presence of a home caregiver to predict their need for extended care after a THA [13].

### Randomization

One hundred patients will be randomized with a one-to-one treatment allocation to the intervention (n=50) or standard care (n=50) group at the time of consent. Allocation concealment in the order of recruitment will be conducted “off-site” after consent has been obtained. Permuted block randomization will be conducted to ensure an equal number of participants are allocated to each group per week. This will be conducted in weekly blocks of 10. Due to the nature of the research, blinding will not be possible for either the participants or the health care team. The researcher conducting the data analysis, however, will be blinded.

### Standard Care

The comparator for the trial, namely standard care, is an Enhanced Recovery Program based on an orthopedic recovery program established in The United Kingdom [14]. It consists of participants receiving paper-based information, attending a 1-hour hospital-based face-to-face pre-operative education session, and a postdischarge follow-up phone call. The education session is presented by a registered nurse, occupational therapist, pharmacist, and physiotherapist and participants are encouraged to bring their support person to the session. Examples of the content in the paper-based information and education session include fall prevention, wound care, showering, healthy eating, returning to work, pain management, and selecting a personal alarm. The postdischarge phone call is conducted within one week of discharge by a registered nurse from the ward. During the phone call the nurse will ask specific questions about how the patient is coping postdischarge, including questions about pain management, wound healing, exercise and mobility, nutrition and hydration, bowel management, and how well the patient is managing daily living activities.

### Intervention

The intervention for the trial is standard care as described above plus the addition of the eHealth education program. The eHealth program was developed specifically for patients undergoing a THA by a multidisciplinary team of clinicians, researchers, and consumer representatives. It provides information about the care requirements during the journey of a hip replacement, advice on well-being, and an exercise program. The information in the eHealth education program is for patients, support persons, relatives, and community health professionals and gives information on what to expect pre-operatively, whilst in hospital, postoperatively, and postdischarge.

**Figure 1:**
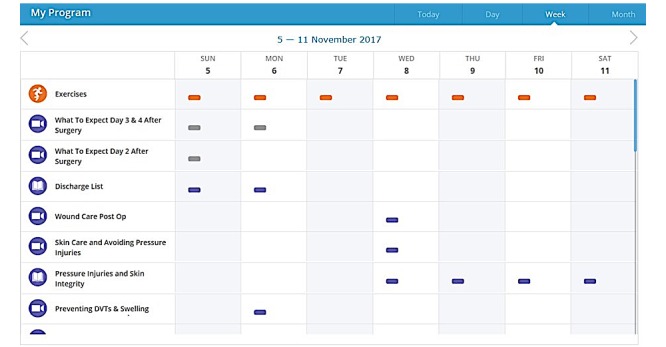
Screenshot example of patient’s “My Program” for one week.

Participants will access the eHealth program via a weblink after registration using a username and password. The program is designed to give participants continual access to information, at a time that suits them without them having to come to the hospital. It is recommended that participants use the program on a daily basis starting 2 weeks prior to surgery until 30 days postsurgery. However, the participants may have access to the program up to 4 weeks pre-operatively and will continue to have access until 12 months postsurgery. The eHealth program provides a suite of educational resources including fact sheets, videos, exercise videos, and email reminders about the pre- and postoperative care of a hip replacement. The resources are linked to the participants’ journey with a focus on personal wellbeing and its importance in the pre-operative and postoperative periods. Some examples of the resources included in the eHealth program are preparation for surgery, nutrition, pre- and postoperative exercise regimes, and medication management with additional information provided on walking aids and safety if living with pets. Each day, when the participant logs into their “My Program” window, it will display a list of videos and information as well as exercises that have been allocated for them to view or complete that day (Figure 1).

Each exercise is allocated a specific number of repetitions and sets that the participant is required to perform, and this information is included with the information about the exercise for the participant. Participants have access to the health care team via the program’s email for 4 weeks postdischarge and can invite their regular health care providers (such as their community occupational therapist and physiotherapist) or support person to be part of the program. The health professional team at the hospital can personalize the participant’s program including the exercises by editing in number of sets and repetitions recommended for each exercise.

Intervention fidelity will be assessed using analytics built into the website. After a participant watches a video or reads information, they will be prompted rate it. The program will then mark it as read and record information about how long the participant spent on that piece of information. In addition, after the exercise videos are completed by the participant, they will be prompted to mark them as complete. Automatically generated emails from within the program are sent on a regular basis to inform participants of the information available on the platform and provide instructions pre- and postoperatively. The health care team have access to the platform and can view the website analytics for each participant.

### Outcome Assessment

**Figure 2:**
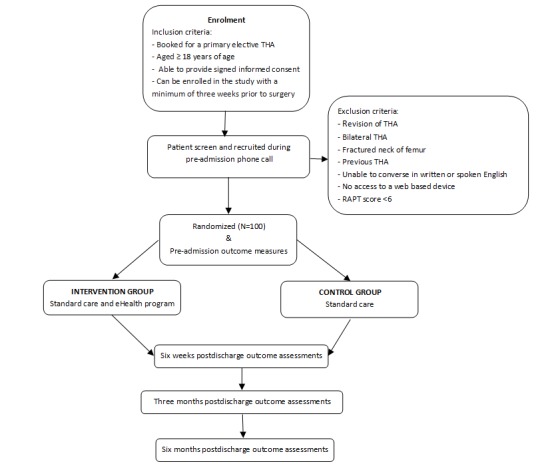
Flow chart of recruitment and trial participation. RAPT: risk assessment and prediction tool; THA: total hip arthroplasty.

Outcome measures will be collected at four time points, namely at pre-admission, 6 weeks, 3 months and 6 months after the surgery. Figure 2 presents a flow chart of the recruitment and trial participation process and Table 1 outlines the outcome assessments used in this study, as well as the collection time points for the assessments. Participants will be asked to complete the relevant assessments online and an email reminder will be sent to each participant when the assessment is due, followed by a reminder one week later.

**Table 1:** Summary of primary and secondary outcome assessments and collection time points used in the study.

Outcome assessment	Pre-admission	6 weeks postdischarge	3 months postdischarge	6 months postdischarge
HOOS^a^	✓	✓	✓	✓
EQ-5D-5L^b^	✓	✓	✓	✓
SEMCD^c^	✓	✓	✓	✓
Satisfaction survey		✓		
Web-based analytics^d^				✓

^a^HOOS: Hip Dysfunction and Osteoarthritis Outcome Score.

^b^EQ-5D-5L: EuroQol EQ-5D-5L.

^c^SEMCD: Self-Efficacy for Managing Chronic Disease.

^d^eHealth program only.

#### Primary Outcome

The primary outcome is patient-reported evaluation of symptoms and function limitations related to the affected hip (the hip undergoing surgery). The measure of this outcome is the Hip Dysfunction and Osteoarthritis Outcome Score (HOOS) version LK 2.0. The HOOS is a validated 40-item questionnaire where patients self-assess their hip across 5 subscales: (1) symptoms and stiffness, (2) pain, (3) function of daily living, (4) sports and recreational activities function, and (5) quality of life [15].

#### Secondary Outcomes

The secondary outcomes measures are quality of life, self-efficacy, patient satisfaction, and Web-based analytics. These will be assessed using the tools listed below.

The EuroQol EQ-5D-5L assessment will be utilized in this study. It is a 5-level 5-dimension standardized assessment tool, used to measure health-related quality of life. The 5 dimensions include: mobility, self-care, usual activities, pain or discomfort, and anxiety or depression. The tool includes a visual analogue scale where participants are asked to rate their health on a scale from 0 to 100, where 0 means the worse health you can imagine and 100 means the best health you can imagine [16].

The Self-Efficacy for Managing Chronic Disease (SEMCD), is a validated tool consisting of six items on a 10-point scale measuring a participant’s level of confidence in doing certain activities, 1 being “not at all confident” and 10 being “totally confident” [17].

Patient satisfaction with the program will be explored using a patient satisfaction survey developed by the research team. The developed survey comprises of 6 closed questions and 2 open questions for participants in both arms of the trial. Participants in the eHealth program will receive additional survey questions on the usability of the app, consisting of 13 closed questions and 3 opened-ended questions.

For participants in the eHealth program additional data on their utilization of the eHealth educational program will be collected through Web-based analytics. This will include information on the modules accessed, length of time spent on each module, and satisfaction ratings prompted within the program.

### Economic Evaluation

A cost-effectiveness analysis comparing the developed eHealth program to standard care will be conducted from the hospital’s perspective on an intention-to-treat basis. Direct costs will include the service delivery costs associated with each program (brochures, photocopying, and Web-based application fees) as well as the cost of follow-up care (the time spent by health professionals organizing and providing face-to-face sessions, replying to emails, making telephone calls, and managing and monitoring online content). Cost data will be collected from pre-admission through to 6 months postdischarge using hospital medical records. Outcomes will include changes in HOOS and quality of life (measured using the EQ-5D-5L). Incremental cost-effectiveness ratios (comparing the difference in cost between the eHealth program and standard care with the difference in the outcomes) will be calculated for HOOS and Quality Adjusted Life Years, and sensitivity analysis will be conducted on the key parameters.

### Sample Size

Sample size calculations were conducted based on the primary outcome, the HOOS. Existing data were used to determine the minimal clinically important improvement [18] and SD [19]. Based on a power of 90% and 5% significance level, 42 patients per group are needed. To allow for a dropout rate of approximately 15%, a sample size of 50 per group is required. Therefore, the estimated required sample size for the study is 100 participants.

### Statistical Analysis

Data will be reported in accordance with the Consolidated Standards of Reporting Trial (CONSORT). Mean (SD), median (interquartile range) and percentages will be used to describe the characteristics of the study group.

An analysis of covariance will be used to assess the primary outcome. The covariables will include age, gender, type of surgery, length of stay, RAPT score, and other comorbidities. Treatment effects will be calculated on the pre-to-post intervention outcomes at 6 weeks. Further analysis will be performed on the posttreatment effects at 3 months and 6 months postsurgery. The clinical treatment effect of each intervention group will be further analyzed using independent *t* tests, tests of medians, nonparametric tests, and chi-squared tests on pre-to-post intervention changes across the range of outcome measures and patient satisfaction.

## Results

Enrollment in the study commenced in January 2018 with recruitment due for completion towards the end of the year. We expect to achieve our goal of 100 participants. The first results are expected to be submitted for publication in 2019.

## Discussion

The outcomes and cost of using an eHealth program to support a patient’s recovery from a THA will be compared with standard care in this RCT. If the eHealth program is found to be effective, implementation into clinical practice could lead to further improvements in patient outcomes. Additionally, the findings of the study will support further research in the use of the eHealth program for other orthopedic surgical procedures such as knee replacements. If the intervention is found to be cost-effective to the hospital, it could support resource efficiency.

Patients scheduled for a revision of THA, a bilateral THA, THA following a fractured neck of femur, or those who have had a previous THA are not included in the trial as their outcomes are likely to be different from those receiving a primary elective THA. A potential limitation of the trial is that in addition to patient self-reported outcome measures, more objective measures of function may have benefitted the trial (eg, the 10-meter walk test and 6-minute walk test). These tests are not included in the study due to the difficulty of conducting these tests pre- and postoperatively, particularly with patients living a distance from the hospital.

The findings from this study will contribute to the current body of literature on eHealth programs in orthopedic care and inform other health professionals on the outcomes of using an eHealth program.

## Abbreviations

CONSORT: Consolidated Standards of Reporting Trial
eHealth: electronic health
EQ-5D-5L: EuroQol EQ-5D-5L
HOOS: Hip Dysfunction and Osteoarthritis Outcome Score
RAPT: risk assessment and prediction tool
RCT: randomized controlled trial
SEMCD: Self-Efficacy for Managing Chronic Disease
THA: total hip arthroplasty
